# Transient Nature of Long-Term Nonprogression and Broad Virus-Specific Proliferative T-Cell Responses with Sustained Thymic Output in HIV-1 Controllers

**DOI:** 10.1371/journal.pone.0005474

**Published:** 2009-05-12

**Authors:** Samantha J. Westrop, Nadeem A. Qazi, Jeffrey Pido-Lopez, Mark R. Nelson, Brian Gazzard, Frances M. Gotch, Nesrina Imami

**Affiliations:** 1 Department of Immunology, Imperial College London, Chelsea & Westminster Hospital, London, United Kingdom; 2 Department of HIV/GU Medicine, Imperial College London, Chelsea & Westminster Hospital, London, United Kingdom; University of Toronto, Canada

## Abstract

**Background:**

HIV-1^+^ individuals who, without therapy, conserve cellular anti-HIV-1 responses, present with high, stable CD4^+^ T-cell numbers, and control viral replication, facilitate analysis of atypical viro-immunopathology. In the absence of universal definition, immune function in such HIV controllers remains an indication of non-progression.

**Methodology/Principal Findings:**

CD4 T-cell responses to a number of HIV-1 proteins and peptide pools were assessed by IFN-γ ELISpot and lymphoproliferative assays in HIV controllers and chronic progressors. Thymic output was assessed by sjTRECs levels. Follow-up of 41 HIV-1^+^ individuals originally identified as “Long-term non-progressors” in 1996 according to clinical criteria, and longitudinal analysis of two HIV controllers over 22 years, was also performed. HIV controllers exhibited substantial IFN-γ producing and proliferative HIV-1-specific CD4 T-cell responses to both recombinant proteins and peptide pools of Tat, Rev, Nef, Gag and Env, demonstrating functional processing and presentation. Conversely, HIV-specific T-cell responses were limited to IFN-γ production in chronic progressors. Additionally, thymic output was approximately 19 fold higher in HIV controllers than in age-matched chronic progressors. Follow-up of 41 HIV-1^+^ patients identified as LTNP in 1996 revealed the transitory characteristics of this status. IFN-γ production and proliferative T-cell function also declines in 2 HIV controllers over 22 years.

**Conclusions:**

Although increased thymic output and anti-HIV-1 T-cell responses are observed in HIV controllers compared to chronic progressors, the nature of nonprogressor/controller status appears to be transitory.

## Introduction

HIV-1 infection typically manifests as a chronic progression to AIDS during which fully functional CD8^+^ cytotoxic T-lymphocyte (CTL) responses are gradually lost [1], [2]. Lack of maintenance of CTL function is at least in part dependent on loss of numbers and function of antigen-specific CD4^+^ helper T lymphocytes (HTL) [2]–[5]. Despite successful suppression of viral replication by highly active antiretroviral therapy (HAART), HIV-1-specific effector T-cell function fails to be reconstituted [6].

In HIV-1^+^ individuals viral replication, disease progression and opportunistic infection inversely correlate with fully functional virus-specific HTL and CTL responses [2], [7]. The term “long-term non-progressor” (LTNP) was originally used before the advent of the viral load assay, to describe a clinically non-progressing HIV-1^+^ patient defined by the maintenance of a stable CD4 T-cell count within the normal range (450–1650 cells/µl blood) for many years, in the absence of antiretroviral therapy [2].

Quantification of HIV-1 viraemia revealed that the majority of persons originally defined as LTNPs did not control viral replication, and disease progression in these patients occurred eventually, although less rapid than in typical HIV-1^+^ individuals [8]. However, a small number of LTNPs exhibited suppression of viral replication to levels below the detection threshold of the viral load assay. The control of viral replication occurs in some patients for prolonged periods, and these are referred to as HIV controllers (HIC). From a cohort of ∼5,000 HIV-1^+^ patients at the Chelsea and Westminster Hospital [9] approximately 0.2% of the cohort have been identified as HICs. Here we present data from six HICs who at the time of sample collection mounted robust cytokine-producing, proliferation competent HIV-1-specific CD4 and CD8 T-cell responses.

Many studies include HIV-1^+^ individuals with atypical rates of disease progression, but discrepancy in terminology and inclusion criteria renders it difficult to compare results across studies. The terms “LTNP”, “Elite controller” and “HIV controller” are commonly used, but with varying criteria of definition; the more stringent the criteria applied, the fewer patients included in the study. Inconsistency in the definition of the nonprogressor is illustrated by example papers which use similar terminology to encompass patients with widely differing characteristics. “Nonprogressors” have been defined as patients with lengths of HIV-1 infection of over 14 years [10], more than 12 years [11], more than 4 years, 9 months [12], or an undisclosed length of time [13]. Similar variability is observed in the CD4 counts used to define nonprogressors which in different reports is higher than 500 [10], [11], “normal” (311–1830 cells/µl blood) [12], or 825–1588 cells/µl blood [13]. Viral loads of patients characterised as nonprogressors also vary greatly between cohorts, ranging from <50 copies/ml plasma [13], through <125 [12], <1,000 [10] to 104,000 copies/ml plasma [11]. Individuals who suppress HIV-1 viral load to below detection limit have been reported as immunologically distinct from those with low level viraemia [4], [14], advocating the importance of viral load when defining patient groups.

Preliminary evaluation of both HIV-1-specific CD4^+^ and CD8^+^ T-cell responses in HICs has shown that IL-2- and IFN-γ-producing Th1 responses are good predictors of stable CD4^+^ T-cell counts and delayed disease progression [4], [7]. Understanding the mechanisms of generation and maintenance of fully functional HIV-1-specific peripheral HTL and CTL responses in chronic HIV-1 infection, similar to those observed in HICs, may be of considerable importance for the development of new, and improvement of existing therapies [2], [4], [7], [15]. Novel, effective immunotherapeutic interventions may enable viral control and retard disease progression either in conjunction with or after termination of HAART [2], [15].

Until now, little has been reported on thymic output in adult HICs, however data from long-term asymptomatic adolescents showed that thymic activity was almost normal when compared to age-matched HIV negative controls [16]. Analysis of TREC levels in SIV^+^ macaques revealed a strong correlation between thymic output and non-progression to AIDS [17].

Six HICs were recently identified using the following criteria; HIV-1^+^ with absence of HIV-1 disease, continuous undetectable viraemia and stable CD4 T-cell count within the normal range for over 8 years, despite lack of anti-retroviral therapy [1], [2]. Here we report cross-sectional data of responses to HIV-1, and thymic output from these six HICs compared to six HIV-1^+^ HAART-naïve and eight HAART-treated, chronically infected progressors.

Longitudinal data detailing the changes in anti-HIV-1 responses over time observed in two HICs, diagnosed as HIV-1^+^ for more than 22 years, is presented along with description of proviral *gag* sequencing data from one of these two HICs, at two time points. In addition to this, we also report the current clinical status of a cohort of 41 “LTNPs” initially identified using clinical criteria in 1996.

## Methods

### Study subjects and samples

Blood samples were collected from patients belonging to the Chelsea and Westminster cohort (Table 1). Up to date clinical information from 41 “LTNP” originally identified in 1996 was compiled. Informed written consent and ethical approval was obtained from the Riverside Research Ethics Committee for the studies described (RREC1108). PBMC were isolated by density gradient centrifugation [4], [18].

**Table 1:** Detailed description of characteristics of the study cohorts.

Patient ID	Sex	CD4 Count, cells/μl blood	CD8 Count, cells/μl blood	HIV-1 RNA, copies/ml plasma	HAART duration, months	Time since HIV-1^+^ diagnosis, years	Age, years
HIC 1	M	722	916	<50	None	8.4	44.8
HIC 2	M	977	946	<50	None	15.2	37.6
HIC 3	M	850	665	<50	None	16.5	51.6
HIC 4	F	1,331	1,107	<50	None	9.1	28.5
HIC 5	M	588	1,225	<50	None	15.9	60.2
HIC 6	M	1,222	1,299	<50	None	7.6	26.9
***Median***		***913.5***	***1,026.5***	***<50***		***12.2***	***41.2***
***(range)***		***(588–1,331)***	***(665–1,299)***	***(<50)***		***(7.6–15.9)***	***(26.9–60.2)***
vCP 1	M	376	751	27,488	None	13.3	51.0
vCP 2	M	665	1,860	3,604	None	9.0	33.7
vCP 3	M	315	1,197	39,271	None	13.7	50.4
vCP 4	M	573	1,423	6,228	None	16.3	35.1
vCP 5	-	590	1,468	12,261	None	-	-
vCP 6	M	700	1,910	27,508	None	16.4	36.7
***Median***		***581.5***	***1,445.5***	***19,874.5***		***13.7***	***36.7***
***(range)***		***(315–700)***	***(751–1,910)***	***(3,604–39,271)***		***(9.0–16.4)***	***(33.7–51.0)***
aCP 1	M	590	1115	<50	14	1.7	30.6
aCP 2	M	442	1080.0	<50	12	1.1	41.5
aCP 3	M	801	1319	<50	64	19.6	57.5
aCP 4	M	838	872	<50	97	19.8	53.1
aCP 5	M	995	1959	<50	113	22.7	52.6
aCP 6	M	503	1701	<50	102	11.6	64.3
aCP 7	M	464	1436	<50	51	20.1	43.6
aCP 8	M	1247	972	<50	93	11.7	43.9
***Median***		***696***	***1217***	***<50***	*78*	***15.6***	***48.2***
***(range)***		***(442–1247)***	***(872–1959)***	***(<50)***	*(12–113)*	***(1.1–22.7)***	***(30.6–64.3)***
P value	-	0.0152*	0.0931*	-	-	0.6623*	0.9307*
		0.2284^#^	0.1812^#^			0.4908^#^	0.3450^#^
		0.2448†	0.5728†			0.8329†	0.2222†
							
HIC 2-2^‡^	M	1,027	1,424	<50	None	21.8	44.2
HIC 2-3^§^	M	895	1,142	<50	None	22.2	44.6
HIC 5-2^‡^	M	442	288	<50	None	22.5	66.8

- , data not available; P values between *HIC and vCP, ^#^HIC and aCP, †vCP and aCP; bold face indicates statistically significant difference measured to a 95% confidence interval by the Mann-Whitney test; ^‡^2^nd^ or ^§^3^rd^ visit respectively.

### Plasma viral RNA and lymphocyte subset quantification

Plasma viral load and enumeration of CD3^+^, CD4^+^ and CD8^+^ T lymphocytes was conducted as described previously [4], [18].

### HIV-1 antigens and peptides

Recombinant HIV-1 antigens: rGag p24 (EVA620), rTat (ARP672) and rEnv gp120 (EVA646) (all baculovirus derived), rNef (EVA650; Bru strain expressed in *E.coli*) and rRev (ARP663.2; from clade B Han2 isolate expressed in *E.coli*) were used in cell culture at a final concentration of 10 µg/ml. The 11 Rev, 8 Tat, 20 Nef and 22 Gag p24 peptides were 20-mers overlapping by 10aa and used at a final concentration of 5 µg/ml (all NIBSC, Potters Bar, UK).

### Proliferation assays

Lymphocyte proliferation of freshly isolated PBMC was measured as described previously [4]. Results are expressed as stimulation index (SI); the experimental result divided by the background (negative control; PBMC and media only). A positive response is defined as SI≥3.

### IFN-γ ELISpot assay using HIV-1 overlapping-peptide stimuli

IFN-γ ELISpot was carried out as described previously on freshly isolated PBMC [4], [18]. Negative controls (cells only) resulted in ≤16 spot forming cells (SFC)/10^6^ PBMC. The positive threshold for IFN-γ production was defined as ≥20 SFC/10^6^ PBMC after subtracting background [18]. Results from triplicate wells were averaged with <10% variation between duplicates.

### PCR analysis of sjTRECs to determine Thymic output

DNA from 5×10^6^ PBMC was extracted using the Puregene DNA purification kit (Gentra, Flowgen, Staffordshire, UK). PCR amplification [19], and quantification of sjTRECs were performed as described previously [20], [21].

### Proviral gag sequencing

Genomic DNA was extracted from 5×10^6^ PBMC using the Nucleon Genomic DNA Extraction Kit (Tepnel Life Sciences) according to the manufacturer's instructions. DNA was stored at 50 ng/µl in RNAse/DNAse free water (Sigma) at −20°C until required. Proviral *gag* was amplified by nested PCR as described previously [22], and the resulting 1.45 kbp product was sequenced using ABI 3730*xl* and ABI 3130*xl* hardware.

### Statistical analysis

GraphPad Prism v5.0 (San Diego, USA) was used for statistical calculations. The Mann-Whitney test was used to compare responses between patient groups, and the Wilcoxon signed rank test was used to compare paired responses from the same patient as appropriate. Significance was measured to a 95% confidence interval (p<0.05).

## Results

### Transient nature of “LTNP” status

**Figure 1:**
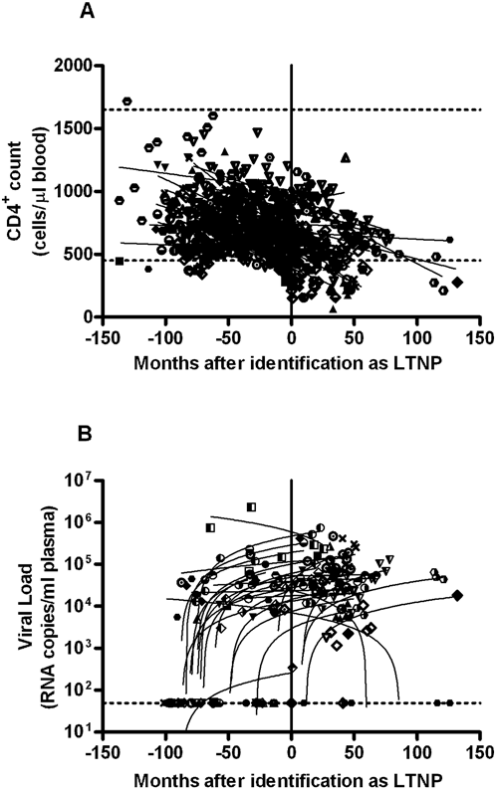
Follow up of 41 “LTNPs” over 132 months since original identification using clinical criteria in 1996. (a) All available pre-HAART data of CD4 T-cell counts for the cohort of 41 LTNPs since identification (normal range 450–1650 cells/µl blood, marked with horizontal line). (b) All available pre-HAART data of plasma viral load for the cohort of 41 LTNPs since identification. DL = detection limit of assay. Data from each individual are plotted with different symbols and regression lines indicate slopes. Once the patient initiates anti-retroviral therapy their characteristics are no longer shown on the graphs.

Follow-up of a cohort of 41 HIV-1^+^ individuals originally defined as “LTNP” in 1996 and identified according to clinical criteria: CD4 count, length of infection and lack of opportunistic infection, was undertaken [1]. Pre-HAART CD4 counts and VL of these patients are presented in Figure 1. Over the course of eleven years, all 41 patients no longer met HIC status, with detectable viraemia and falling CD4 counts, and the entire cohort is now being prescribed HAART – which concurs with studies described by others [8].

### Sustained HIV-1 Tat-, Rev- and Nef-Specific T-Cell Responses in HICs

**Figure 2:**
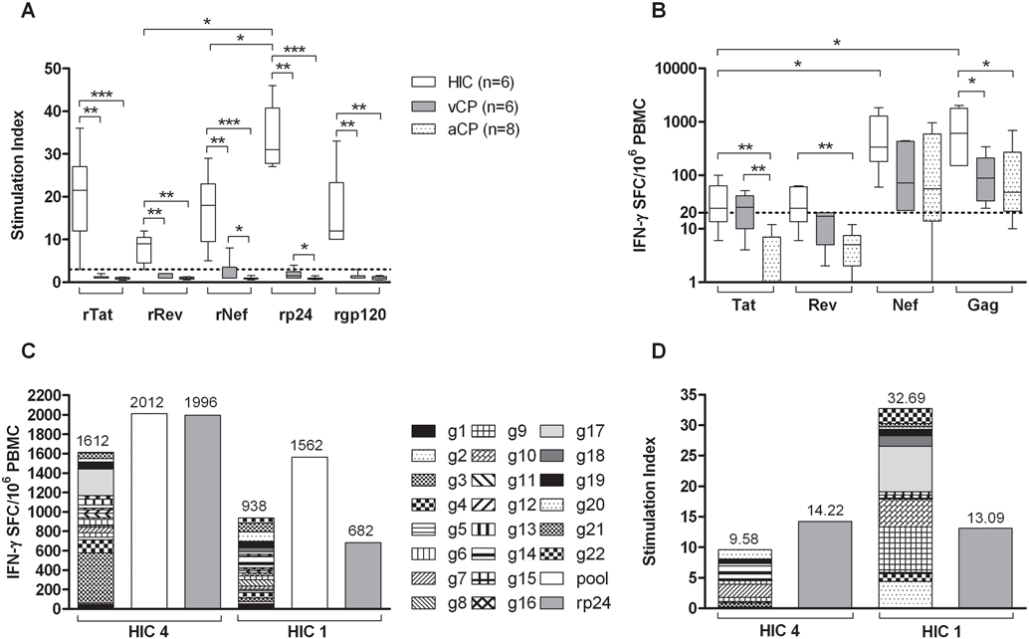
Significantly higher lymphoproliferative responses to HIV-1 recombinant antigens mounted by HIV controllers (HIC) compared to HIV-1^+^ viraemic chronic progressors (vCP) and aviraemic chronic progressors (aCP) despite comparable IFN-γ release to HIV-1 peptide pools. Broad IFN-γ and proliferative responses to HIV-1 Gag p24 in HICs. Box plots show the median with IQR indicated. Whiskers represent the 10^th^–90^th^ percentiles. The Mann-Whitney test was used to compare responses between patient groups, and the Wilcoxon signed rank test to compare paired responses from the same patient. *p<0.05, **p<0.01, ***p<0.001. (a) Lymphoproliferative response to rTat, rRev, rNef, rp24 (Gag) and rgp120 (Env), from HICs (n = 6), vCPs (n = 6) and aCPs (n = 8). The threshold for positivity (SI ≥3) is marked. (b) IFN-γ release (spot forming cells per million PBMC), from HICs (n = 6), vCPs (n = 6) and aCPs (n = 8), in response to stimulation with Tat, Rev, Nef and Gag p24 overlapping peptides. Data represent mean values of triplicate wells with <10% variation among triplicates. The threshold of 20 SFC/10^6^ PBMC is marked. (c) Summation of IFN-γ release in response to stimulation with individual overlapping Gag p24 peptides (1–22) compared to the response to the peptide pool and rp24 protein. Data from two representative HICs is shown. (d) Summation of lymphoproliferative responses, of two representative HICs, to stimulation with individual overlapping Gag p24 peptides (1–22) compared to the response to rp24 protein.

Significantly higher proliferative responses to HIV-1 Tat, Rev, Gag, Env and Nef (all p<0.01) recombinant antigens were observed in the six HICs compared to 6 viraemic and 8 aviraemic chronic progressors (Figure 2A). The three patient cohorts were age- and length of infection-matched (detailed in Table 1). In HICs the proliferative responses to rTat, rNef, rRev and rEnv were similar, however the responses to rRev and rNef were significantly lower than those resulting from stimulation with rGag (p = 0.0313) (Figure 2A). This may be due to the lower antigenic load of regulatory Rev and Nef compared to the capsid protein Gag p24. Although close to the threshold of positivity, both the anti-rRev and -rNef proliferative responses in HICs were above negative cut-off and statistically significantly higher than the responses mounted by HAART-naïve, viraemic chronic progressors (vCPs; p = 0.0022 and p = 0.0043 respectively) and HAART-treated aviraemic chronic progressors (aCPs; p = 0.0024 and p = 0.0007 respectively. Figure 2A). All proliferative responses mounted by aCPs in response to the HIV-1 antigens tested were below the threshold of positivity (SI<3). The significantly higher IFN-γ responses to Tat peptides (Figure 2B), and proliferative responses to rNef and rp24 (Figure 2A) by vCPs compared to aCPs may be due to the lower antigenic burden in aCPs who are receiving successful HAART. It is of note however that only 1 CP (vCP 2) mounted a positive proliferative response which was only just above threshold (rp24 SI = 4, rNef SI = 8). All other CPs mounted no proliferative responses to any of the HIV-1 antigens tested.

In addition to proliferation, IFN-γ was released when PBMCs from HICs were stimulated with Tat and Rev peptides as well as Gag and Nef peptides (Figure 2B). IFN-γ release in vCPs was significantly lower than HICs when Gag peptides were used (p = 0.0411), but similar with Tat, Rev and Nef peptide stimuli. Aviraemic CPs (n = 8) on successful HAART (Table 1) exhibited significantly lower IFN-γ production in response to Tat peptides from the responses mounted by the vCPs and HICs, presented in Figure 2 (both p values <0.01). These results demonstrate a distinction between the IFN-γ producing and proliferative ability of HIC CD4 T cells and the IFN-γ-only responses observed in chronic progressors [23], [24], which does not appear to be dependent on HIV-1 plasma viraemia as aCPs, with HAART-suppressed viral load, as well as vCPs, fail to mount a lymphoproliferative response to HIV-1 antigens.

### Cumulative response to individual p24 Gag peptides

Stimulation with Gag peptides or recombinant protein elicited the dominant anti-HIV-1 response in HICs, concurring with previous data showing an inverse correlation of anti-Gag responses to viral burden [25]. The IFN-γ response (median: 611 SFC/10^6^, range: 152–2,012) was significantly higher in HICs than vCPs (p = 0.041) and HAART-treated aCPs (p = 0.0168; Figure 2B). The proliferative response to rGag (median: SI 34.5, range: 27–65) was significantly higher than in vCPs (p = 0.002) and aCPs (p = 0.0007; Figure 2A). Consequently the anti-Gag response was investigated further.

The sum of the IFN-γ responses to 22 individual p24 overlapping peptides was similar to that resulting from stimulation with the Gag peptide pool and rGag antigen (Figure 2C). Additionally, the sum of the lymphoproliferative responses to individual peptides was similar to the proliferation that occurred in response to rp24 (Figure 2D). These data suggest functional internalisation, processing and presentation of exogenous antigen by antigen presenting cells of these HICs. In contrast, previously published data shows that the proliferative responses to individual overlapping Gag peptides were absent in a cohort of chronically infected HIV-1^+^ patients [4].

### Thymic output

**Figure 3:**
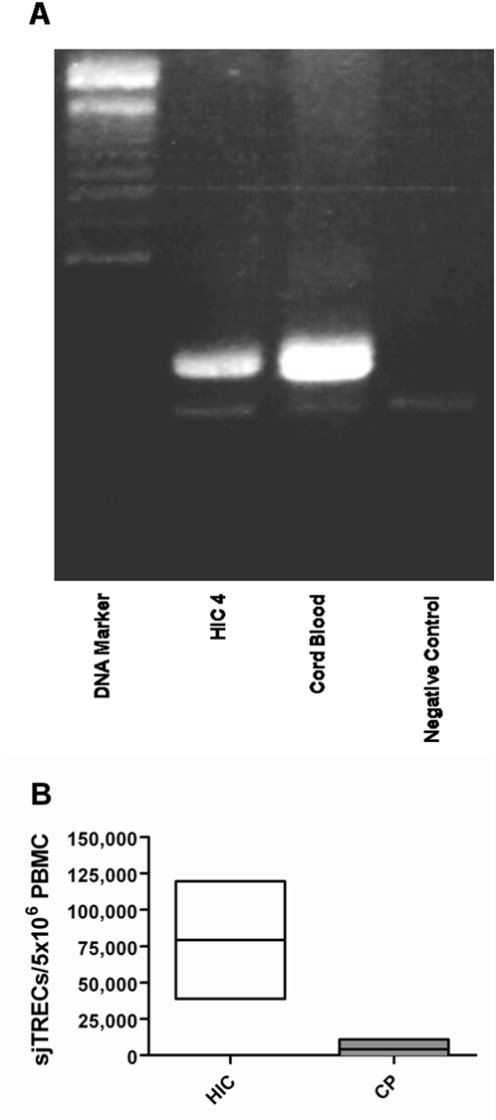
Higher levels of thymic output in HIV controllers (HIC) compared to age-matched chronic progressors (CP). (a) Electrophoresis gel showing an example of TREC levels in PBMC of HIC4 compared to the positive control, cord blood. (b) TREC levels in PBMC of 6 HICs compared to previously published data of PBMC from chronic progressors [26]. The two cohorts are age-matched (p = 0.3823; median age HIC 41.2 years, range 26.9–60.2; median age CP 36.5 years, range 20.2–57.7). The CD4 count was significantly higher in HICs than HAART naïve CPs (p<0.0001; HIC median 913.5 cells/µl blood, range 588–1331; CP median 254.5 cells/µl blood, range 13–551).

TREC levels were detectable in the PBMCs of all six HICs and levels of TRECs observed in cord blood lymphocytes were used as a positive control (Figure 3A). TREC levels in the six HICs ranged from 38,817 to 119,586 TRECs/5×10^6^ PBMC with an average TREC level of 79,201.5/5×10^6^ PBMC – approximately 19 fold higher than previously reported by ourselves in HAART-naïve chronic progressors, using the same methodology, where only 48% had detectable TREC levels (average: 4,200 TRECs/5×10^6^ PBMC, range: −2,463–10,863) (Figure 3B) [26]. The comparative cohort of chronic progressors were age-matched to the six HICs (p = 0.3823; median age HIC 41.2 years, range 26.9–60.2; median age chronic progressor 36.5 years, range 20.2–57.7) and the CD4 count was significantly higher in HICs than HAART-naïve CPs (p<0.0001; HIC median 913.5 cells/µl blood, range 588–1331; CP median 254.5 cells/µl blood, range 13–551).

### Reduction in T-cell-mediated anti-HIV-1 responses in two HICs

Two HICs identified as HIV-1^+^ in 1984 and 1986 were assessed for anti-HIV-1 T-cell-mediated responses at two and three time points (respectively). Patient HIC 5 donated blood to our studies at 191 and 270 months (15.9 and 22.5 years respectively) after HIV-1^+^ diagnosis (Table 1). Blood samples were obtained from patient HIC 2 at 182, 262 and 267 months (15.2, 21.8 and 22.2 years respectively) post-HIV-1^+^ diagnosis (Table 1).

**Figure 4:**
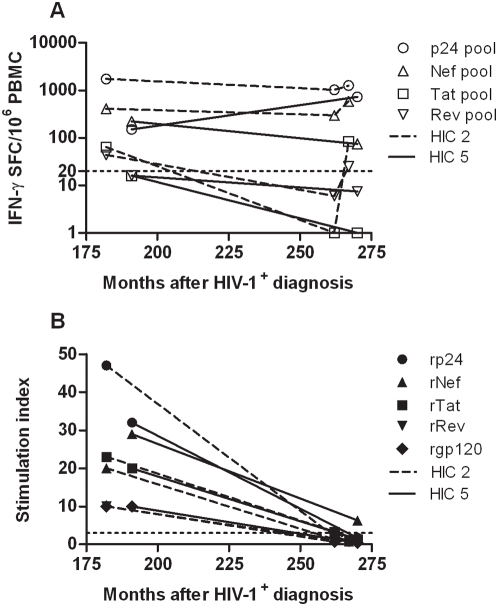
Longitudinal analysis of T-cell mediated responses, to HIV-1 antigens and peptides, by two HIV controllers over a 79 and 85 month period. Data is shown from two HICs; HIC 5 and HIC 2, from two and three time points respectively. Data represent mean values of triplicate wells with <10% variation among triplicates. (a) IFN-γ release (spot forming cells/10^6^ PBMC) in response to stimulation with Tat, Rev, Nef and Gag p24 overlapping peptides. The threshold of 20 SFC/10^6^ PBMC is marked. (b) Lymphoproliferative response to rTat, rRev, rNef, rp24 (Gag) and rgp120 (Env). The threshold for positivity (SI ≥3) is marked.

IFN-γ release in responses to overlapping HIV-1 Tat, Nef, Rev and Gag peptide pools, and proliferative responses to recombinant HIV-1 Tat, Nef, Rev, Gag and Env proteins are shown in Figure 4A and B respectively.

The IFN-γ response mounted by HIC 2 to Gag and Nef peptide pools remained relatively constant over the three time points, spanning 85 months (7.1 years), and the IFN-γ responses to Tat and Rev peptides decreased below positive threshold at month 262, but were then regained five months later (Figure 4A). In contrast, the proliferative responses to all of the recombinant HIV-1 proteins declined to below positive threshold for the assay (SI of 3) (Figure 4B). Patient HIC 5 also exhibited a loss of proliferative response to rGag, rTat and rEnv, but maintained a positive proliferative response to rNef, albeit greatly reduced (∼22% of previous proliferative response) (Figure 4B). The IFN-γ responses mounted by HIC 5 were largely conserved, with partial decrease to Tat, Rev and Nef peptide pools, and an increase in response to Gag (Figure 4A).

### Proviral p17 and p24 gag sequencing in an HIV Controller

Proviral p17 and p24 were sequenced from the PBMC of patient HIC 2 at 262 and 267 months post HIV-1^+^ diagnosis. The sequence was compared to the HXB2 consensus sequence using the online HIV database [www.hiv.lanl.gov]. At time point 262 months the sequence of proviral *gag* in the patient was 96% similar to HXB2. Five months later the HIV-1 proviral *gag* sequence was 92% similar to HXB2, indicating that despite undetectable viral load (<50 copies HIV-1 RNA/ml plasma), changes in proviral sequence occurred, suggesting residual viral replication.

## Discussion

Here we present data from a cohort of strictly defined HICs. Proliferative and IFN-γ responses in HICs were assessed by stimulating PBMC with a range of HIV-1 proteins and peptides. We describe HICs who currently retain both functional IFN-γ and lymphoproliferative responses to regulatory Tat, Rev and Nef, in addition to structural Gag and Env. In contrast, HIV-1-specific lymphoproliferative responses were absent in both viraemic and HAART-suppressed aviraemic HIV-1^+^ chronic progressors. The presence of multi-functional anti-HIV-1 Nef-, Tat- and Rev-specific T cells suggests Nef, Tat and Rev as potential additional antigenic targets that correlate with viral control. Gag- and Nef-specific T cells may differ, with a higher proportion of Gag-specific effector-memory T cells displaying stronger survival potential, however, presence of both proliferation competent and IL-2 producing HIV-1 Gag- and Nef-specific T cells have been observed in controllers with stable CD4 counts [4].

Successful stimulation of proliferation of CD4^+^ T cells with recombinant proteins, demonstrates the retention of functional antigen internalisation, processing and presentation, as well as the proliferative ability of CD4^+^ T cells in these HICs. Proliferative and IFN-γ responses of HICs to rGag p24 were shown to be comparable to stimulation with individual 20 aa peptides, also indicating functional presentation of exogenous antigen.

The preserved, functional response to the early stage protein Tat appears to suggest a lack of Tat-associated viral escape in these HICs, probably resulting from initiation and maintenance of immune control at an early stage of infection. Anti-Rev and -Nef responses mounted by the HICs, although significant, were considerably lower than responses to other viral antigens tested. This may be due to the lower antigenic burden of Rev and Nef, compared to Gag, when viral load is undetectable. This is supported by the report of a positive correlation between plasma viral load and the number of anti-Rev and -Nef responses [25]. It is also of note that an overzealous anti-Rev response may exert selection pressure which could lead to reduced Rev activity. Consequently Gag and Env expression could be lowered, reducing sensitivity to anti-Gag and -Env CTL responses [27], and possibly contributing to the increased viral load and consequent disease progression observed in chronically infected HIV-1^+^ individuals.

The evidence that 41 previously defined LTNPs have, since initial identification in 1996, all progressed, suggests that despite maintaining clinical nonprogression for a number of years, “LTNP” status is not necessarily permanent [1] [8]. Consequently the salient issues to address are why and through which process nonprogressor status is lost. We have observed in HICs that prior to progression, there is a loss of anti-HIV-1 response [4]. Anti-Env and -Nef responses appear to diminish first, with no apparent immediate affect on viral load [4]. It appears that the subsequent loss of the multi-functional, balanced anti-Gag response accompanied by loss of virological control, may result in increased viraemia. These observations concur with the reported association of anti-Gag responses with low viraemia, and responses to Env and accessory and regulatory proteins (including Nef) with increased viral load [3], [25]. The longitudinal data from two HICs demonstrates a loss of proliferative responses to HIV-1 antigens over time. Fluctuation in IFN-γ production to the peptides of regulatory proteins Tat and Rev is observed in both patients, which may be attributable to the loss of the proliferative ability of Tat- and Rev-specific CD4 T cells. However, it is of note that both of these patients, to date, still suppress HIV-1 plasma load to below detection limit, have normal CD4 T-cell counts and are not receiving HAART. It remains to be seen whether the proliferative responses originally observed 7–8 years ago will be reinitiated in the future, or if these patients are on the verge of disease progression.

In addition to the demonstration of the dominance and breadth of anti-Gag responses in HICs, the observation that the presence/absence of an anti-p24 response appears to be the watershed between viral control and progression reiterates the importance of p24 Gag as an immunological target [4], [25].

A multitude of host factors connected to disease progression are reviewed in [28], however specific, unanimous definition of the LTNP or the HIC remains to be established. As well as creation of universal criteria for defining nonprogressors, further detailed immunogenetic studies are necessary to investigate why such patients eventually lose HIV-1-specific responses and progress to chronic disease.

Failure of viral control after many years of nonprogression may result from demise in thymic function. Compromised thymic output [29], and a correlation of CD4 T-cell recovery following ART initiation and thymic volume [30], has been reported in chronic HIV-1^+^ infection. This is in contrast with normal thymic function in HICs presented here. Thymic atrophy, which occurs with advancing age in healthy individuals, may play a role in the eventual loss of viral control that has been observed in the cohort of 41 “LTNP”, due to the fact that after ∼20 years of HIV-1 infection the cohort is an ageing one (median age 50.5 years, range 40.5–71.8 years). The lower levels of sjTRECs/5×10^6^ PBMC, previously observed in chronic progressors, may not be due solely to a reduction in thymic function, but also dilution of sjTRECs, resulting from immune hyperactivation-induced proliferation in these individuals [31], [32]. However, the ratio between sjTRECs (generated after many rounds of intrathymic proliferation) and βTRECs (generated early in thymocyte maturation) indicates the extent of intrathymic proliferation [33], which determines the thymic output of naïve T cells [34]. In previous studies, this analysis of ratios has confirmed that sjTREC levels provide a good indication of thymic output, and are not hugely influenced by peripheral proliferation [32], [33]. Nevertheless the reliability of measuring sjTRECs to infer thymic output has not yet been unanimously accepted. Another point to consider when interpreting the TREC data presented here is the higher number of CD4 T cells in HICs compared to CPs. The causality of higher TREC levels in HICs compared to CPs is therefore debateable, and may be due to either increased thymic output replenishing the naïve T-cell pool or retention of CD4 T-cells once they are produced or most likely both. It has also recently been shown that the *in vivo* lifespan of effector T-cell subsets is reduced in HIV-1^+^ chronic progressors, compared to healthy controls [35], and it is possible that the half life of HIV-1-specific effector T cells in HICs is longer than in chronic progressors. This ability to preserve HIV-1-specific effector T cells may not be permanent, and a reduction in lifespan of T cells may precede disease progression.

Another possible reason for eventual disease progression is a breach in the gut-blood barrier, leading to microbial translocation and subsequent immune hyperactivation, as described in acute chronic infection [36]. This breach may result from a decline in gut cell/tissue regeneration and consequent alteration of the environment [36]. Both of these areas warrant further investigation.

The levels of HIV-1 proviral DNA in HICs have been shown to be approximately 7 times lower than in chronic progressors [24]. Despite undetectable plasma viraemia and lower levels of integrated provirus, sequencing of HIV-1 proviral *gag* in an HIC revealed changes in the DNA sequence over time, which may be indicative of low level viral turnover [37]. Potential low level replication, along with the reduction of the proliferative response to rp24 in this patient, between these two time points, advocates the possibility that eventually the virus may escape from the immune control of HICs. However further data on proviral HIV-1 sequences is necessary before any conclusion can be drawn.

The cause and effect relationship of viral load and effective anti-HIV-1 responses remains debatable. The inability of HIV-1-specific CD8 T cells to proliferate [7], and load lytic granules is thought not to be a direct consequence of viral load, as lack of proliferative and cytolytic potential are not reversed by effective HAART-mediated suppression of viral load in chronic progressors as shown here, and previously described [38], [39].

Cross-sectional, and (ideally) longitudinal, studies of atypical patients, such as the HICs presented here, are crucial for the elucidation of the correlates of progression in HIV-1 disease. By deciphering what constitutes an effective immune response, we will know what to endeavor to achieve with therapeutic intervention.
